# Anti-HFRS Human IgG Produced in Transchromosomic Bovines Has Potent Hantavirus Neutralizing Activity and Is Protective in Animal Models

**DOI:** 10.3389/fmicb.2020.00832

**Published:** 2020-05-07

**Authors:** Casey C. Perley, Rebecca L. Brocato, Hua Wu, Christoph Bausch, Priya P. Karmali, Jerel B. Vega, Melanie V. Cohen, Brandon Somerville, Steven A. Kwilas, Lucia M. Principe, Joshua Shamblin, Padmanabh Chivukula, Eddie Sullivan, Jay W. Hooper

**Affiliations:** ^1^Virology Division, United States Army Medical Research Institute of Infectious Diseases, Frederick, MD, United States; ^2^SAB Biotherapeutics Inc., Sioux Falls, SD, United States; ^3^Arcturus Therapeutics Inc., San Diego, CA, United States

**Keywords:** hantavirus, hantaan, puumala, hamster, marmoset, HFRS, transchromosomic bovine, passive transfer

## Abstract

We explored an emerging technology to produce anti-Hantaan virus (HTNV) and anti-Puumala virus (PUUV) neutralizing antibodies for use as pre- or post-exposure prophylactics. The technology involves hyperimmunization of transchomosomic bovines (TcB) engineered to express human polyclonal IgG antibodies with HTNV and PUUV DNA vaccines encoding G_n_G_c_ glycoproteins. For the anti-HTNV product, TcB was hyperimmunized with HTNV DNA plus adjuvant or HTNV DNA formulated using lipid nanoparticles (LNP). The LNP-formulated vaccine yielded fivefold higher neutralizing antibody titers using 10-fold less DNA. Human IgG purified from the LNP-formulated animal (SAB-159), had anti-HTNV neutralizing antibody titers >100,000. SAB-159 was capable of neutralizing pseudovirions with monoclonal antibody escape mutations in G_n_ and G_c_ demonstrating neutralization escape resistance. SAB-159 protected hamsters from HTNV infection when administered pre- or post-exposure, and limited HTNV infection in a marmoset model. An LNP-formulated PUUV DNA vaccine generated purified anti-PUUV IgG, SAB-159P, with a neutralizing antibody titer >600,000. As little as 0.33 mg/kg of SAB-159P protected hamsters against PUUV infection for pre-exposure and 10 mg/kg SAB-159P protected PUUV-infected hamsters post-exposure. These data demonstrate that DNA vaccines combined with the TcB-based manufacturing platform can be used to rapidly produce potent, human, polyclonal, escape-resistant anti-HTNV, and anti-PUUV neutralizing antibodies that are protective in animal models.

## Introduction

Hantaan virus (HTNV) and Puumala virus (PUUV) are zoonotic viruses, causing a disease known as hemorrhagic fever with renal syndrome (HFRS) (Elliot et al., 2013). Hantaviruses, family *Hantaviridae*, have a tripartite negative-sense RNA genome, and infection with these viruses can result in HFRS characterized by varying degrees of capillary leakage, acute kidney injury, and hemorrhage (Elliot et al., 2013). Each year tens of thousands of cases of HFRS are reported world-wide, with a case fatality rates between 1 and 15% (Elliot et al., 2013). Most HFRS cases caused by HTNV and related viruses are in China and the Korean peninsula, with PUUV cases predominantly in Scandinavia and western Russia (Jonsson et al., 2010; Zhang et al., 2010; Elliot et al., 2013). There are currently no FDA-licensed vaccines or therapeutics for disease caused by any hantavirus (Schmaljohn, 2009; Brocato and Hooper, 2019).

The M-segment of hantaviruses encode viral envelope glycoproteins (G_*n*_ and G_*c*_) (Cifuentes-Munoz et al., 2014). DNA vaccines encoding the M-segment of numerous hantavirus species have demonstrated immunogenicity in a wide variety of animal species and humans (Hooper et al., 1999, 2001a, 2006, 2008, 2014b; Custer et al., 2003; Boudreau et al., 2012; Brocato et al., 2012; Bounds et al., 2015; Haese et al., 2015). Passive transfer of DNA-vaccine derived antibodies was efficacious in preventing disease or infection against their cognate virus in hamster models of infection or disease (Custer et al., 2003; Hooper et al., 2008, 2014a; Brocato et al., 2012; Haese et al., 2015). For HTNV specifically, passive transfer of sera from rhesus macaques vaccinated with a HTNV DNA M-segment vaccine protected hamsters against infection with HTNV (Custer et al., 2003).

Transchromosomic bovines (TcB) produce antigen-specific, fully human, immunoglobulins (IgG) in addition to chimeric antibodies (human γ heavy chain and bovine κ light chain) depending on the type of TcB (Sano et al., 2013). After vaccination against a particular target, fully human polyclonal IgG against the cognate antigen can be purified from large volumes of plasma (Hooper et al., 2014a). TcB have been used to produce polyclonal neutralizing antibody products against numerous viruses including Zika virus, Ebola virus (EBOV), Venezuelan Equine Encephalitis and Middle East respiratory syndrome coronavirus, and two hantaviruses that cause hantavirus pulmonary syndrome, Andes virus (ANDV) and Sin Nombre virus (SNV) (Hooper et al., 2014a; Bounds et al., 2015; Dye et al., 2016; Luke et al., 2016; Gardner et al., 2017; Stein et al., 2017). For all of these viruses, passive transfer of TcB-derived IgG immediately prior to or post challenge prevented disease, elicited partial protection, or rapidly reduced viral titer (Hooper et al., 2014a; Bounds et al., 2015; Dye et al., 2016; Luke et al., 2016; Stein et al., 2017; Rosenke et al., 2018). Here, we demonstrate that it is possible to combine DNA vaccine technology with the TcB platform to produce potent human polyclonal IgG for use as a pre- or post-exposure prophylactic for HFRS caused by HTNV and PUUV infection.

## Materials and Methods

### Ethics

Animal research was conducted under an IACUC approved protocol at SAB Biotherapeutics (USDA Registration Number 46-R-0012). Animal research was conducted under an IACUC approved protocol at USAMRIID (USDA Registration Number 51-F-00211728 & OLAW Assurance Number A3473-01) in compliance with the Animal Welfare Act and other federal statutes and regulations relating to animals and experiments involving animals. The facility where this research was conducted is fully accredited by the Association for Assessment and Accreditation of Laboratory Animal Care, International and adheres to principles stated in the Guide for the Care and Use of Laboratory Animals, National Research Council, 2011.

### DNA Vaccines

The HTNV M segment-based DNA vaccine [pWRG/HTN-M(co)] was previously described (Hooper et al., 2001a). This HTNV M segment DNA vaccine sequence was used to generate the single and double escape mutants from the literature (Wang et al., 1993; Kikuchi et al., 1998). The Seoul virus (SEOV) M segment-based DNA, pWRG/SEO-M(opt2) is identical to the previously published pWRG/SEO-M(x) vaccine (Hooper et al., 1999), however, the open reading frame was optimized for human codon usage and mRNA stability (Fath et al., 2011). The PUUV DNA vaccine, pWRG/PUU-M(s2), SNV DNA vaccine, pWRG/SN-M(opt), and ANDV DNA vaccine, pWRG/AND-M(opt2), have been published previously (Brocato et al., 2013; Hooper et al., 2013; Kwilas et al., 2014). The Choclo virus (CHOCV) DNA vaccine, pWRG/CHOCV-M(opt) and Dobrava virus (DOBV) DNA vaccine, pWRG/DOB-M(opt), were constructed by cloning an optimized synthetic M gene open reading frame into the Not 1-BglII site of pWRG7077. All M segments were generated at Genewiz unless otherwise noted. Research grade plasmids used to vaccinate TcB were manufactured by Aldevron (Fargo, ND, United States).

### Plasmid LUNAR^®^ Formulation

The formulations for lipid-based delivery were prepared by mixing an Arcturus-propriety combination of lipids dissolved in ethanol with an aqueous phase containing plasmid DNA dissolved in citrate buffer (pH 3.5) using a Nanoassemblr microfluidic device (Precision Nanosystems, Vancouver, BC, Canada). The molar percentage ratio for the constituent lipids is 58% ATX (proprietary ionizable amino lipids), 7% DSPC (1,2-distearoyl-sn-glycero-3-phosphocholine) (Avanti Polar Lipids), 33.5% cholesterol (Avanti Polar Lipids), and 1.5% DMG-PEG (1,2-Dimyristoylsn-glycerol, methoxypolyethylene glycol, PEG chain molecular weight: 2,000) (NOF America Corporation). The solutions were combined in the microfluidic device at a flow ratio of 1:3 ethanol:aqueous phases. The total combined flow rate was 12 mL/min, per microfluidics chip. The mixed material was then diluted with phosphate buffer after leaving the micromixer outlet. The diluted particles were purified by dialysis in Hepes buffer (pH 7.4) containing sucrose using regenerated cellulose membranes (SpectraPor Tube-A-Lyzers, Spectrum Laboratories). A total of 200 diavolumes were exchanged, effectively removing ethanol and ensuring complete buffer exchange. The LNPs were then concentrated using ultra-spin centrifugal filter units (Amicon Ultra Centrifugal Filter Units, EMD Millipore). Particle size was determined by dynamic light scattering (ZEN3600, Malvern Instruments). DNA content and encapsulation efficiency were determined using ribogreen assay. Encapsulation efficiency was calculated by determining unencapsulated plasmid DNA content by measuring the fluorescence upon the addition of ribogreen (Molecular Probes) to the particles (Fi) and comparing this value to the total plasmid DNA content that is obtained upon lysis of the particles by 1% Triton X-100 (Ft), where % encapsulation = (Ft − Fi)/Ft × 100. A potency assay was implemented to confirm the formulated material was potent enough for vaccination. Briefly, a potency assay involves a 96-well plate of HEK293T cells plated at 1E5 cells/well 24 h prior to assay. A non-formulated plasmid using 2ug of DNA and Fugene 6 is used as the standard for transfection. The formulated material is run at 2 ug. Half-log dilutions are made of the Fugene/unformulated plasmid mixture and the formulated material being tested as it is self-transfecting and does not require normal transfection setup steps. After 18–24 h, cells are fixed and stained using specific rabbit anti-sera. The well counted for potency has fluorescent focus units (ffu) between 20 and 200 green cells. This then is converted to ffu/ug. A plasmid must be at least 500 ffu/ug to be deemed potent.

### Transchromosomic Bovines (TcB)

The TcB used in this study (#2034 and #2026) are homozygous double knockouts in endogenous bovine heavy chain immunoglobulin genes (IGHM^–/–^, IGHML1^–/–^) and contain the human artificial chromosome (HAC) vector cKSLHACD (Sano et al., 2013; Matsushita et al., 2014). The TcB #2303 is homozygous triple knockouts in endogenous bovine immunoglobulin genes (IGHM^–/–^, IGHML1^–/–^, and IGL^–/–^) and contain the human artificial chromosome (HAC) vector KcHACD (Sano et al., 2013; Matsushita et al., 2014, 2015). The HAC cKSLHACD and KcHACD contain a fragment of human chromosome 14, the human immunoglobulin heavy chain locus except the IgHM constant region, which remains bovine, and human chromosome 2, the entire human immunoglobulin κ light chain locus (Sano et al., 2013; Matsushita et al., 2014, 2015).

### Vaccination of TcB

TcB #2034 was vaccinated four times intramuscularly with unformulated pWRG/HTN-M(co) DNA vaccine (12 mg/vaccination) with SAB proprietary adjuvant SAB-adj-1. The DNA vaccine was delivered using the PharmaJet Stratis device at four injection sites behind the left and right ear and on the left and right hind leg (1 mg/injection, 3 mg/injection site). SAB-adj-1 was delivered as a 1.5 mL injection using needle and syringe adjacent (1–2 cm) to the PharmaJet Stratis DNA-vaccination site. TcB #2026 was vaccinated with a low-dose lipid nanoparticle-formulated pWRG/HTN-M(co) DNA vaccine (1.2 mg/vaccination), via the PharmaJet Stratis injection device at four injection sites behind the left and right ear and on the left and right hind leg (0.3 mg/injection, 0.3 mg/injection site). TcB #2303 was vaccinated with an lipid nanoparticle-formulated pWRG/PUU-M(s2) DNA vaccine (1.2 mg/vaccination), via the PharmaJet Stratis injection device similar to TcB #2026 vaccination.

### Purification of Anti-HFRS Tc Human IgG

Purification of human IgG from TcB has been previously described previously (Kuroiwa et al., 2009; Hooper et al., 2014a). The purified anti-HTNV Tc human IgG SAB-159 used in neutralization and animal studies is derived from plasma drawn after the fourth vaccination and has a concentration of 50.48 mg/mL. The purified anti-PUUV Tc human IgG SAB-159P used in neutralization and animal studies is derived from plasma drawn after the third, fourth, and fifth vaccinations and has a concentration of 40.11 mg/mL. As a negative control purified Tc human IgG from a non-immunized cow was provided at 48.38 mg/mL. All purified IgG is in sterile liquid containing 10 mM glutamic acid monosodium salt, 262 mM D-sorbitol and 0.05 mg/mL Tween_80_ (pH 5.5).

### Viruses, Cells, Medium, and Antibodies

Hantaan virus strain 76–118 (Lee et al., 1978) and PUUV strain K27 (Tkachenko et al., 1984) were propagated in Vero E6 cells (Vero C1008, ATCC CRL 1586) in T-150 flasks and collected from infected-monolayer supernatants. Cells were maintained in Eagle’s minimum essential medium with Earle’s salts containing 10% fetal bovine serum (FBS), 10 mM HEPES (pH 7.4), 1× penicillin-streptomycin, amphotericin B (0.5 μg/ml) and gentamicin sulfate (50 μg/ml) at 37°C in a 5% CO_2_ incubator. Cell debris was removed by low speed centrifugation in a table top centrifuge. HTNV and PUUV were twice plaque purified according to published methods (Hooper et al., 2001b). Virus stocks were aliquoted and stored at −60°C or colder. Virus identity has been confirmed by sequencing of the stocks. MAbs 3D5 and HCO2 were obtained from the Joel Dalrymple collection at BEI Resources.

### Animal Procedures

Female Syrian hamsters (*Mesocricetus auratus*) 6–8 weeks of age (Envigo, Indianapolis, IN), or adult marmosets (*Callithrix jacchus*) weighing over 300 g were anesthetized by inhalation of vaporized isoflurane using an IMPAC6 veterinary anesthesia machine. Once anesthetized, animals were injected with the indicated concentration of virus diluted in PBS. Intramuscular (i.m.) (caudal thigh) injections consisted of 0.2 ml delivered with a 1 ml syringe with a 25-gauge, 5/8 in needle. Subcutaneous injections consisted of 2 mL (hamster) or 1 mL (marmoset) of anti-HFRS Tc human IgG diluted in PBS administered with a 3 ml syringe with a 23-gauge, 1 in needle at a single injection site. Vena cava blood draws occurred under previously stated methods of anesthesia, and were limited to 7% of total blood volume per week. Terminal blood collection was performed by heart stick at the time of euthanasia. All work involving animals was performed in an animal biosafety level 3 (ABSL-3) laboratories.

### ELISA

The enzyme-linked immunosorbent assay (ELISA) used to detect N-specific antibodies (N-ELISA) was described previously (Elgh et al., 1997). Species-specific secondary antibodies were used at the following concentrations: peroxide-labeled anti-hamster (1:10,000) (Sera Care, Gaithersburg, MD, United States), and alkaline phosphatase conjugated anti-monkey (1:1,000) (MilliporeSigma, St. Louis, MO, United States). Assays using peroxide labeled antibodies were developed with TMB microwell peroxidase substrate at an absorbance of 450 nm. A sample was considered positive if its peak optical density (OD) value was greater than either 0.025 or the background value (the average of three negative control wells + 3 times their standard deviation, whichever was higher. The specific OD sum is the summation of all values greater than background and represents the area under the ELISA titer curve.

### Plaque Reduction Neutralization Test (PRNT)

Plaque reduction neutralization test (PRNT) were performed as previously described with minor modifications (Schmaljohn et al., 1985; Chu et al., 1995; Boudreau et al., 2012). HTNV-infected monolayers were fixed 7 days post-infection by 2 mL of 10% formalin per well. Immunostaining was performed as previously described (Hooper et al., 2014b). Plaques were stained using 3D7-conjugated to HRP (1:1,000) (Life sciences) eliminating the need for a secondary antibody. All sera samples were assayed in duplicate beginning at a 1:20 dilution.

### Pseudovirion Production

Pseudovirions were produced using the HTNV, SEOV, PUUV, DOBV, ANDV, SNV, and CHOCV DNA vaccine plasmids described above. HEK293T cells were seeded in T75 tissue culture flasks and transfected with the plasmid of interest using Transporter 5 (Polysciences Inc) at ∼80% confluency. After ∼18 h the transfection media was removed and the cells were infected with VSVΔG^∗^rLuc at a multiplicity of infection of ∼0.02 for 1 h at 37°C. The media was removed and fresh media was added, the flasks were then incubated at 32°C for 72 h. The supernatant from infected cells was collected and clarified by high speed centrifugation, followed by a PEG 8,000 precipitation with 3.2% salt. The PEG mixture is spun at10K xG for 45 min. The pellet was resuspended overnight in 1 mL TNE buffer, then filtered using a 0.45 μm filter, aliquoted and stored at 70°C.

### Pseudovirion Neutralization Assay (PsVNA)

The PsVNA utilizes a replication-restricted, recombinant vesicular stomatitis virus (rVSV^∗^ΔG) expressing luciferase, which is pseudotyped with the hantavirus glycoproteins-of-interest. HTNV DNA plasmids containing the H304Y, K795Q, and H304Y/K795Q double mutation were manufactured by Genewiz. The PsVNA used to detect neutralizing antibodies in sera was described previously (Hooper et al., 2014a; Kwilas et al., 2014). First, heat-inactivated sera were diluted 1:10, followed by five-fold serial dilutions that were mixed with an equal volume of Eagle’s minimum essential medium with Earle’s salts containing, 10% fetal bovine sera (Gibco), 1% Pen-Strep (Gibco), and 10% human complement (Cedarlane) containing 4,000 fluorescent focus units of pseudovirions. This mixture was incubated overnight at 4°C. Following this incubation, 50 μl was inoculated onto Vero cell monolayers in a clear bottom, black-walled 96-well plate in duplicate. Plates were incubated at 37°C for 18–24 h. The media was discarded and cells were lysed according to the luciferase kit protocol (Promega, Madison, WI, United States). A Tecan M200 Pro was used to acquire luciferase data. The values were graphed using GraphPad Prism (version 7) and used to calculate the percent neutralization normalized to cells alone and pseudovirions alone as the minimum and maximum signals, respectively. The percent neutralization values for duplicate serial dilutions were plotted. Eighty percent PsVNA (PsVNA_80_) and 50% PsVNA (PsVNA_50_) titers were interpolated from 4-parameter curves, and geometric mean titers were calculated. NAU for this study were calculated by multiplying the PsVNA_80_/mL x volume (mL) used.

### Statistical Analysis

Details for each statistical test completed are described (Supplementary Table 1). Briefly, unpaired *t*-tests, two-tailed were conducted for, N-ELISA titers for comparisons of hamsters of the same viral challenge dose (Figure 3E), and marmoset N-ELISA specific OD sum comparisons between treatment and control groups (Figure 4B). Ordinary one-way ANOVA with multiple comparisons test was conducted on N-ELISA titers on remaining figures (Figures 3, 5). Analyses were conducted using GraphPad Prism (version 7).

## Results

### TcB Vaccination Responses

**FIGURE 1:**
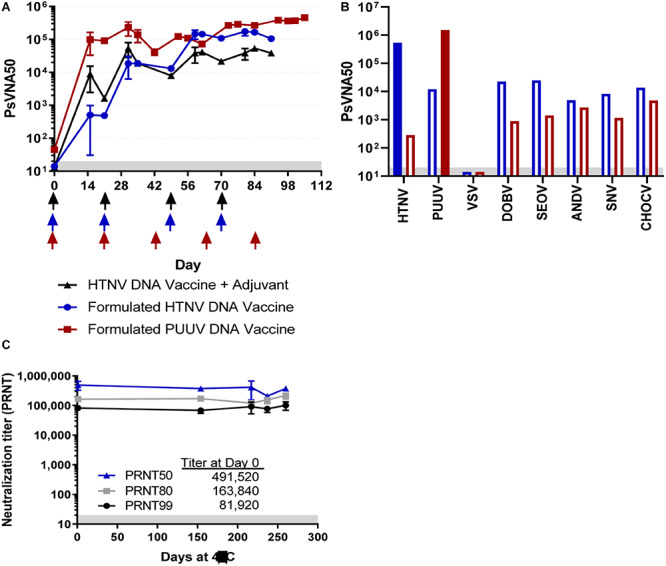
Neutralizing antibody responses in TcB vaccinated with either HTNV or PUUV DNA vaccines. **(A)** TcB #2034 (black symbols) was vaccinated with unformulated HTNV DNA vaccine and a veterinary adjuvant at the times indicated by the black arrows, TcB #2026 (blue symbols) was vaccinated with a LMP-formulated HTNV DNA vaccine at the times indicated by the blue arrows, and TcB #2303 (red symbols) was vaccinated with a LNP-formulated PUUV DNA vaccine at the times indicated by the red arrows. Sera was collected from the TcB at the indicated timepoints and represents the geometric mean titer ± standard deviation of 2–5 independent assays (Days 14, 21, 56, and 84, all others represent data from a single assay). **(B)** SAB-159 and SAB-159P were evaluated for neutralizing (solid bars) and cross neutralizing (open bars) activity by PsVNA. **(C)** SAB-159 purified material stored at 4°C for the indicated number of days was evaluated by PRNT. Limit of quantitation is 20 (gray shaded area).

Previous experiments to produce anti-ANDV and anti-SNV TcB human IgG have demonstrated that including an SAB-adj-1 adjuvant at the injection sight increased immunogenicity of the ANDV and SNV DNA vaccines resulting in higher titer virus-specific neutralizing antibodies (Hooper et al., 2014a). More recently we found that LNP formulation increased the efficiency of DNA vaccine immunogenicity in multiple species, including TcB (manuscript submitted). Here we initially compared the response elicited by a HTNV M segment based DNA vaccine using either SAB-adj-1 adjuvant or LNP formulation. One TcB (#2034) was vaccinated with 12 mg DNA using the PharmaJet Stratis^®^ needle-free disposable syringe jet injection device. SAB-adj-1 adjuvant was administered by needle at the site of DNA injection. This is similar to the vaccination strategy used previously, except here adjuvant was delivered with each vaccination instead of just the final boost (Hooper et al., 2014a). A second TcB (#2026) was vaccinated with LNP-formulated HTNV DNA vaccine at a lower dose (1.2 mg per vaccination), using the PharmaJet Stratis^®^ injection device (Figure 1A). Serum samples collected throughout the vaccination series were analyzed for neutralizing antibody titers using a pseudovirion neutralization assay (PsVNA). Both bovines developed neutralizing antibodies against HTNV at the first blood collection timepoint, which was 2 weeks after the first dose. While TcB #2034 initially had higher neutralizing titers (PsVNA_50_ ∼16 fold higher on Day 14 and ∼fourfold higher on Day 31), TcB #2026 ultimately yielded higher titers beginning after the third vaccination (PsVNA_50_ ∼fourfold higher on day 70 and ∼fivefold higher on day 91). Anti-HTNV Tc human IgG (hence forth referred to as SAB-159) was purified from plasma collected 2 weeks after the fourth vaccination. With the marked increase in HTNV titer using the LNP-formulated vaccine, a third TcB (#2303) was vaccinated with an LNP-formulated PUUV DNA vaccine according to a 5-dose vaccination schedule (Figure 1A). Very high titer anti-PUUV antibody was detected as early as Day 14, the first timepoint tested. After the last vaccination the PsVNA_50_ titer was well over 100,000. Anti-PUUV Tc human IgG (hence forth referred to as SAB-159P) was purified from plasma following the third, fourth, and fifth vaccination.

### Neutralizing Activity of Purified Human Anti-HFRS Polyclonal Antibodies

SAB-159 (50.48 mg/mL) and SAB-159P (40.11 mg/mL) were evaluated for anti-HTNV and anti-PUUV neutralizing activity by PsVNA. High levels of neutralizing activity were detected for both purified antibody products. PsVNA_50_ titers are plotted in Figure 1B. The PsVNA_50_ and PsVNA_80_ titers of SAB-159 were 540,350 and 296,650, respectively. The PsVNA_50_ and PsVNA_80_ titers of SAB-150P were 1,517,146 and 614,444, respectively. Based on the PsVNA data and protein concentrations of the purified material, the inhibitory concentration 50% (IC_50_) of SAB-159 is 93 ng/mL and the IC_80_ is 170 ng/mL. The IC_50_ of SAB-159P is 26 ng/mL and the IC_80_ is 65 ng/mL. Levels of cross-neutralization against other hantaviruses associated with HFRS or HPS were determined by PsVNA. The PsVNA_50_ values for all heterotypic hantaviruses tested were at least 10-fold lower than both the anti-HTNV and anti-PUUV titers (Figure 1B). SAB-159 was also monitored for stability. No significant change in functional activity, as measured by PRNT, was detected after 8 months at 4°C (Figure 1C).

**FIGURE 2:**
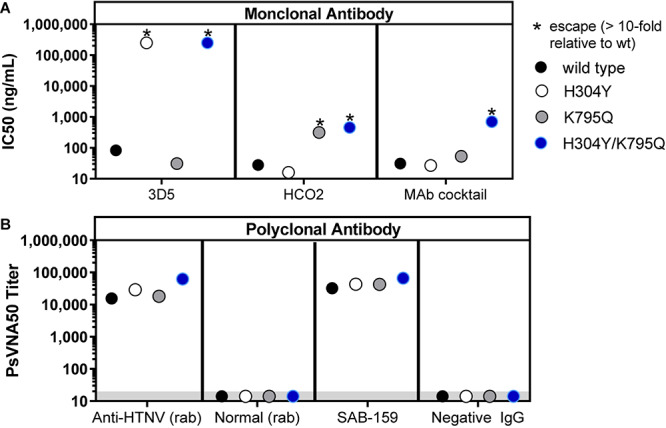
SAB-159 neutralizes MAb escape mutants. Pseudovirions with wildtype G_n_G_c_, single MAb escape mutations, or a double escape mutation were used in PsVNA to determine the effect mutations have on the capacity of antibody to neutralize virion entry into cells. **(A)** Anti-HTNV MAb 3D5, MAb HCO2, and a 1:1 MAb cocktail of those antibodies were evaluated in the PsVNA. The IC50 were plotted. **(B)** The anti-HTNV purified human IgG (SAB-159) prediluted at 1:30 and control polyclonal antibodies were evaluated by PsVNA. The PsVNA_50_ titers were plotted. The positive control was anti-HTNV rabbit (rab) sera; the negative control was normal rabbit sera; and the negative control was purified IgG antibody from a naïve TcB (Negative IgG). *Indicates that the mutation(s) in the GnGc resulted in a >10-fold reduction in PsVNA_50_ titer (escape). Limit of quantitation is 20 (gray shaded area).

To evaluate whether SAB-159 was neutralizing HTNV by binding multiple neutralizing epitopes versus one dominant neutralizing epitope, an experiment was conducted involving pseudovirions engineered to escape known monoclonal antibodies. Neutralizing monoclonal antibody (MAb) escape mutants of HTNV G_n_ and G_c_ have previously been reported (Wang et al., 1993; Kikuchi et al., 1998). Two escape mutations were engineered into the HTNV pseudovirions, individually and in combination. Mutation H304Y in the G_n_ protein escapes MAb-3D5 and mutation K795Q in G_c_ escapes MAb-HCO2. The H304Y, K795Q, and H304Y/K795Q double mutant pseudovirions were used in the PsVNA to evaluate the neutralization escape resistance of SAB-159 (Figure 2). Each monoclonal antibody, alone and in a 1:1 cocktail, were evaluated using the wild-type and mutant pseudovirions (Figure 2A). As predicted, MAb-3D5 neutralized wild-type HTNV pseudovirions and pseudovirions with the MAb-HCO2 escape mutation; however, this antibody did not neutralize pseudovirions with the H304Y mutation or the H304Y/K795Q double mutation where the IC_50_ were >10^5^ ng/mL. Similarly, MAb-HCO2 neutralized wild-type HTNV pseudovirions and pseudovirions with the MAb-3D5 escape mutation; however, MAb-HCO2 failed to completely neutralize pseudovirions with the K795Q mutation or the H304Y/K795Q double mutation (i.e., >10-fold difference in IC_50_ versus wild-type). The 1:1 MAb cocktail neutralized the single escape mutants but only partially neutralized the double mutant. In contrast, SAB-159 was capable of neutralizing wild-type and both single and double neutralization escape mutant pseudovirions (Figure 2B). The positive control anti-HTNV rabbit polyclonal antibody also neutralized all pseudovirions whereas, the negative control rabbit sera or normal TcB purified IgG had no neutralizing activity (Figure 2B). These data demonstrate that the potent neutralizing activity of SAB-159 is targeting multiple epitopes involving both the G_n_ and G_c_ proteins and is resistant to mutations that allow escape from neutralizing monoclonal antibodies.

### Bioavailability of TcB Human IgG in Syrian Hamsters

**FIGURE 3:**
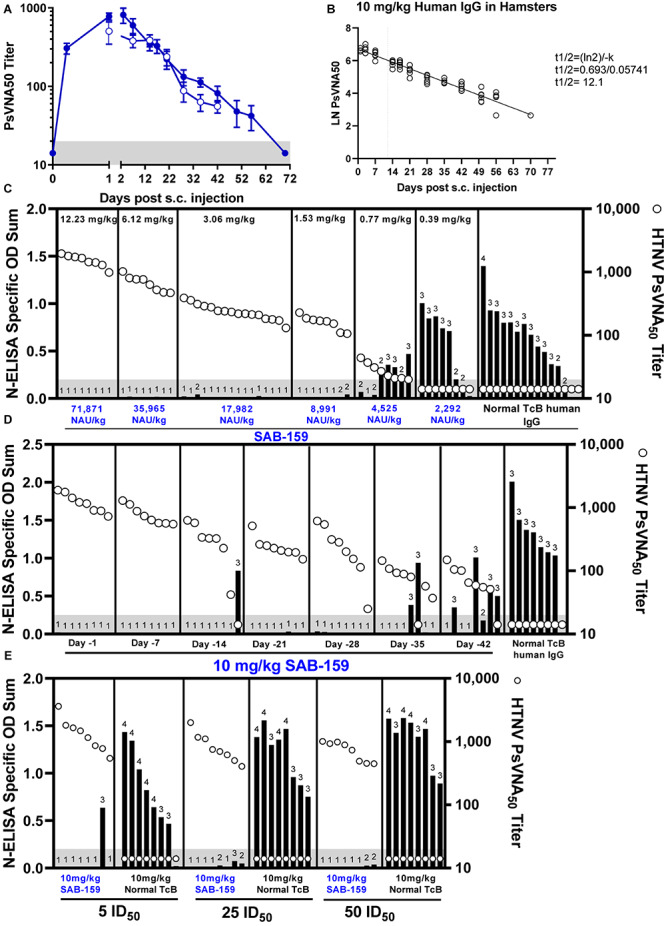
SAB-159 protects hamsters when administered prior to HTNV exposure. **(A)** Hamsters were administered 10 mg/kg SAB-159 subcutaneously and serum collected and analyzed by HTNV PsVNA. The results of two experiments are shown for hamsters that were either serially bled following SAB-159 administration (*n* = 6, closed symbols) or hamsters bled on the indicated day following SAB-159 administration (*n* = 8, open symbols). GMT ± SEM are plotted. **(B)** The data from **(A)** was used to calculate the half-life of 12.1 days based on PsVNA_50_ data (closed symbols used for calculation). **(C)** Characterization of the protective efficacy of SAB-159 against HTNV challenge was determined by N-ELISA when SAB-159 was administered in decreasing concentrations on Day -1 prior to HTNV challenge, **(D)** at 10 mg/kg at increasing timepoints prior to HTNV challenge, and **(E)** at 10 mg/kg with increasing concentrations of HTNV challenge doses. PsVNA_50_ titers (open circles) are shown for hamsters in **(C–E)**. The shaded gray area represents the limit of detection for the PsVNA assay. N-ELISA log_10_ titers are displayed for individual hamsters, ≥2 is positive.

Hantaan virus is highly infectious in Syrian hamsters (ID_50_ = 1.5 PFU) but does not cause disease (Perley et al., 2019). The hamster model was used to evaluate the capacity of SAB-159 to protect against infection. First, SAB-159 was injected subcutaneously at a dose of 10 mg/kg and sera were collected over several weeks. 10 mg/kg was equivalent to a dose of 58,766 neutralizing antibody units (NAU)/kg where a NAU/mL was defined as the PsVNA_80_ titer/mL. Sera were evaluated for neutralizing activity by PsVNA (Figure 3A). Data from the 10-week PsVNA curve was used to calculate an antibody half-life of 12.1 days (Figure 3B).

### Protection From Low-Dose HTNV Infection in Syrian Hamsters

To determine the dose of SAB-159 required to protect against infection, hamsters were administered decreasing concentrations of neutralizing antibody ranging from 12.23 mg/kg to 0.39 mg/kg SAB-159 subcutaneously 1 day prior to a 10 PFU HTNV challenge. Negative control hamsters were treated with 12.23 mg/kg normal TcB human IgG via the same route. Five weeks after challenge infection status was evaluated by anti-N ELISA (Figure 3C). An anti-N response is indicative of a productive hantavirus infection whereas the absence of an anti-N response indicates the animals were protected from infection. Hamsters receiving either 12.23 mg/kg or 6.12 mg/kg SAB-159 were 100% protected from infection (8/8 hamsters seronegative, *p* < 0.0001, see Supplementary Table 1 for additional statistical details), while hamsters receiving 3.06 mg/kg or 1.53 mg/kg SAB-159 were protected 94% and 75% of the time [15/16 (*p* < 0.0001) and 6/8 (*p* < 0.0001) hamsters seronegative, respectively]. A single animal receiving 0.77 mg/kg SAB-159 and no animals receiving 0.39 mg/kg SAB-159 were protected from HTNV infection (*p* = 0.3034 and *p* = 0.9997, respectively). Thirteen of 15 negative control hamsters were infected. This dose ranging experiment demonstrated that a dose as low as 1.53 mg/kg, which was equivalent to 8,991 NAU/kg, could protect against infection in this model.

Next, we designed an experiment to determine how far in advance SAB-159 could be administered and still protect against HTNV challenge (Figure 3D). Beginning on Day -42 and following every week thereafter, groups of hamsters were administered 10 mg/kg SAB-159 by the subcutaneous route. On Day 0, hamsters were challenged with 10 PFU HTNV. All hamsters administered SAB-159 on Days -1, -7, -21, and -28 were protected from infection (*p* < 0.0001for all groups). A single hamster administered SAB-159 on Day -14 was not protected from infection (7/8 animals seronegative, *p* < 0.0001). Of the hamsters administered SAB-159 on Days -35 and -42, 75% and 38% were protected, respectively (*p* = 0.0007 and *p* = 0.1878, respectively). A single negative control hamster was uninfected (7/8 animals infected). This experiment demonstrated that a dose of 10 mg/kg of SAB-159 administration several weeks prior to exposure to HTNV was capable of protecting against infection.

To determine if SAB-159 could protect against higher doses of HTNV, groups of 8 hamsters each were administered 10 mg/kg SAB-159 or normal TcB-derived IgG on Day -1 and then challenged with increasing concentrations of HTNV corresponding to 5 ID_50_, 25 ID_50_, or 50 ID_50_ (Figure 3E). Administration of SAB-159 protected at least 5/8 hamsters in all groups including the highest, 50 ID_50_, challenge dose (*p* < 0.0001). Hamsters administered 10 mg/kg of normal IgG were not protected regardless of challenge dose (23/24 infected). These data demonstrated that SAB-159 at 10 mg/kg could protect against at least 50 times the ID_50_.

For the experiments shown in Figures 3C,D, the level of neutralizing antibody in the sera at the time of challenge was measured by PsVNA. The PsVNA_50_ titers (*n* = 135) combined with the protection status (yes or no), were analyzed by logistic regression to determine the fiducial limits yielding the estimated probability of protective levels of serum neutralizing antibody (Table 1). Results of the logistic regression indicated that log_10_ titer is significantly associated with protection outcome. Each unit log_10_ increase in pre-challenge HTNV PsVNA_50_ titer was associated with an increase in odds of protection against HTNV of 83.0 times (*p* < 0.0001, or 95% CI = 21.3, 323.6). In general, a PsVNA_50_ titer of >100 is predictive of a >80% chance of protection.

**TABLE 1:** Calculation of estimated probabilities of protective immunity against HTNV infection based on protection data from Figure 3.

**Estimated probability of protective immunity^a^**	**HTNV PsVNA_50_**	**Lower 95% fiducial limit**	**Upper 95% fiducial limit**
0.40	41	28	56
0.50	50	35	70
0.60	62	44	89
0.70	78	56	117
0.80	103	73	167
0.90	157	106	293

*^*a*^Determined by logistic regression by Probit. Protection status by titer was used to calculate estimated probabilities of protective immunity.*

### Protection From HTNV Infection in Marmosets

**FIGURE 4:**
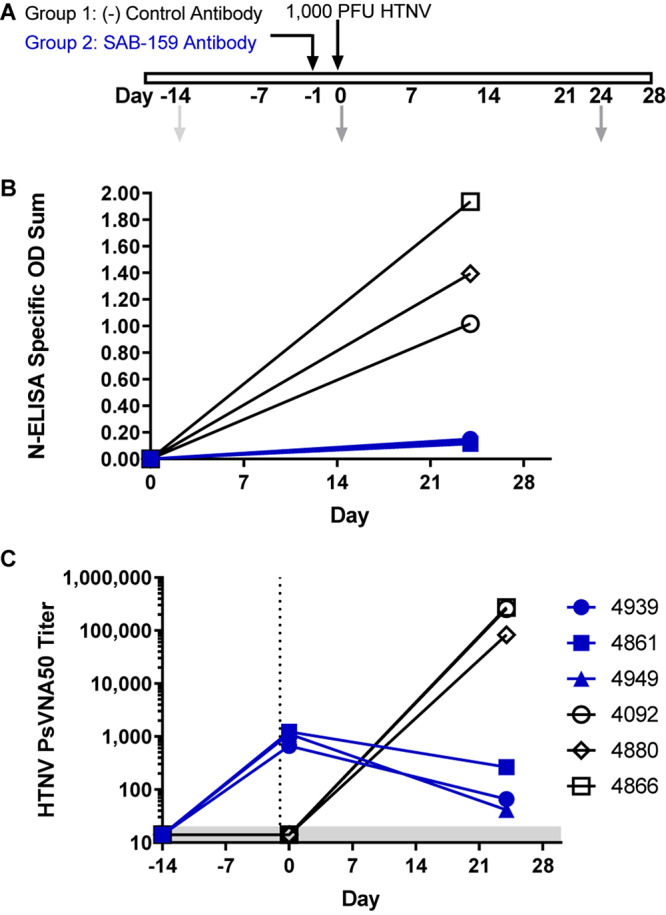
SAB-159 limits HTNV infection in marmosets. **(A)** Experimental design. Two groups of 3 marmosets each were administered either 71,871 NAU/kg (12.23 mg/kg) SAB-159 or control human IgG on Day -1 prior to HTNV challenge. Sera collection dates are shown in gray arrows. Serum from indicated times was analyzed by **(B)** N-ELISA and **(C)** PsVNA. The shaded gray area represents the limit of detection for the PsVNA assay.

Like Syrian hamsters, marmosets can be infected with HTNV as measured by seroconversion but do not develop disease (Perley et al., 2019). Specifically, an intramuscular injection of 1,000 PFU of HTNV resulted in high anti-N by ELISA and high neutralizing antibody titers 30 days after challenge. To test if SAB-159 can protect marmosets from 1,000 PFU of HTNV, animals were administered either 12.23 mg/kg (i.e., 71,871 NAU/kg) SAB-159 or control human IgG subcutaneously 1 day prior to HTNV intramuscular challenge (Figure 4A). Infection status was measured by N-ELISA 24 days after exposure. ELISA O.D. values for the negative control animals increased from <0.1 to 1.02–1.94 indicating that the animals were productively infected. Similarly, the PsVNA_50_ titers for the negative control animals went from below detection on Day -14 and Day 0 to >10,000 on Day 24. In contrast, all marmosets given SAB-159 exhibited minimal change in the N-ELISA O.D. 24 days after challenge indicating infection had been affected by treatment (*p* = 0.0078) (Figure 4B). The animals receiving SAB-159 had PsVNA_50_ titers between 664 and 1,233 on Day 0 representing input neutralizing antibody that was bioavailable (Figure 4C). The PsVNA_50_ titers dropped by Day 24 indicating that the input antibody had waned and that there was no evidence of a productive endogenous immune response in the SAB-159 treated animals. Even a treatment dose of 6,500 NAU/kg (2 mg/kg) SAB-159 remained bioavailable in marmosets 21 days after intraperitoneal injection (Supplementary Figure 1). These data demonstrate that SAB-159 successfully limited infection with HTNV in marmosets.

### Pre- and Post-exposure Treatment of Purified Human Anti-HFRS Polyclonal Antibodies in Hamsters

**FIGURE 5:**
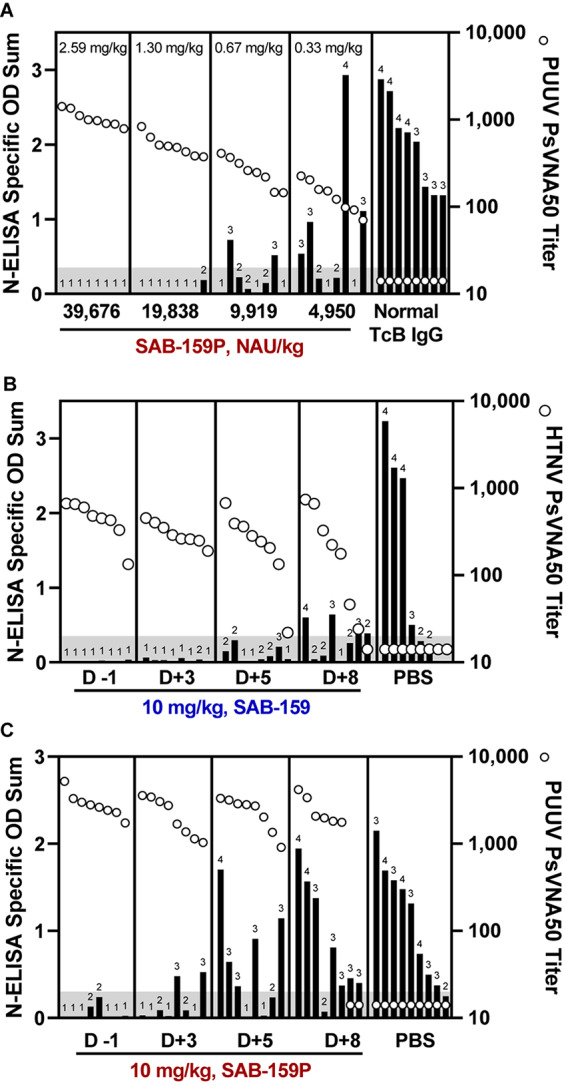
Human IgG products protect hamsters from infection when administered post-exposure. **(A)** Hamsters were administered decreasing concentrations of SAB-159P on Day -1 prior to a 1,000 PFU PUUV challenge. Sera collected on Day 0 was analyzed by PUUV PsVNA (open circles) and Day 35 by N-ELISA (black bars). Characterization of the protective efficacy of **(B)** 10 mg/kg SAB-159 and **(C)** 10 mg/kg SAB-159P administered on indicated days post infection. For **(B,C)**, sera was collected 1 day following passive transfer for analysis in PsVNA (open circles) and Day 35 for analysis in N-ELISA (black bars). **(A–C)** The shaded gray area represents the limit of detection for the PsVNA assay. N-ELISA log_10_ titers are displayed for individual hamsters, ≥2 is positive.

An initial experiment to test the efficacy of the candidate anti-PUUV product, SAB-159P, was conducted with groups of 8 hamsters each receiving descending dosages of SAB-159P ranging from 2.59 mg/kg to 0.33 mg/kg administered 1 day prior to a 1,000 PFU PUUV challenge. Negative control hamsters were administered 2.59 mg/kg of normal TcB IgG. Neutralizing antibody levels in hamster sera on day 0 (day of challenge) were measured by PUUV PsVNA and infection status 5 weeks after challenge was determined by anti-N ELISA (Figure 5A). All hamsters receiving 2.59 mg/kg SAB-159P were protected from infection, 7/8 hamsters receiving 1.30 mg/kg SAB-159P were protected, 3/8 hamsters receiving 0.67 mg/kg were protected and 2/8 hamsters receiving 0.33 mg/kg were protected resulting in statistically significant levels of protection (0.33 mg/kg SAB-159P treatment group, *p* = 0.0067). Eight of 8 negative control hamsters were infected (Figure 5A).

Next, we designed two experiments to determine if SAB-159 and SAB-159P were protective if administered after virus exposure. SAB-159 and SAB-159P, administered at 10 mg/kg corresponding to treatment doses of 58,766 NAU/kg and 153,190 NAU/kg, respectively, were administered on Days -1, +3, +5, and +8 relative to either a 10 PFU HTNV (Figure 5B) or 1,000 PFU PUUV (Figure 5C) challenge. In both experiments, purified human IgG administered on Day +3, but not +5, resulted in a statistically significant level of protection from virus infection (SAB-159 *p* = 0.0022, SAB-159P *p* = 0.0014).

## Discussion

The HTNV and PUUV DNA vaccines used to immunize the TcB in this study are the same vaccine constructs that are currently under evaluation in Phase 1 and 2 clinical trials (Brocato and Hooper, 2019). The anticipated use of those vaccines is as a pretreatment to protect individuals living, traveling or working in areas where PUUV and HTNV are known to cause HFRS. For persons traveling or working in disease-endemic areas, such as military personnel deployed or training in those areas, there might not be sufficient lead time to receive the full vaccine regimen and develop protective immunity. In those cases, receiving passively transferred antibody as a form of instant, albeit relatively short-lived, protective immunity could be a prudent course of action. Such a product would need to be potent enough to require a single injection and, like any pretreatment, would need to have a negligible safety risk.

A potent neutralizing antibody product could also be used as a post-exposure prophylactic for person at very high risk. For example, persons sleeping in the same tent or performing the same work duties as an HFRS patient would be at high risk of exposure. Treatment with a neutralizing antibody product also has the potential to lower viremia and might be effective as a therapeutic to reduce disease severity. In the present study we have demonstrated that the polyclonal neutralizing antibodies are effective when administered pre- and post-exposure but we do not have disease models to obtain post disease onset efficacy.

In ongoing HFRS clinical trials, the DNA vaccines are not LNP-formulated and no adjuvant is used. However, in TcB it is possible to use LNP-formulated DNA or adjuvants approved for veterinary use to increase the immunogenicity of the vaccines. Using LNP-formulated DNA, we were able to produce purified human polyclonal antibody batches with very high neutralizing activity: SAB-159 had an IC_50_ of 93 ng/mL and SAB-159P had an IC_50_ of 26 ng/mL, respectively (Figure 1B). These IC_50_ values were lower than two protective monoclonal antibodies targeting ANDV where the IC_50_ of purified antibodies ranged from 205 to 6,600 ng/mL ANDV (Garrido et al., 2018), and similar to MAb114 (IC_50_ 90 ng/mL), a potent anti-EBOV monoclonal antibody currently in clinical trials as a monotherapy (Corti et al., 2016; Mulangu et al., 2019).

We used the PsVNA titer data to determine IC_50_ and IC_80_ of purified antibody preparations, and we chose PsVNA_80_ to determine NAU. The IC_50_ values are used to compare relative potency for different lots of purified product when the antibody concentration is known. The NAU/mL are used to compare the relative potency of serum, plasma, and purified antibody regardless of whether protein concentration is known. Previously we had used PRNT_80_ titers to determine NAU (Brocato et al., 2012); however, data generated to date indicate that the HTNV PsVNA_80_ and PRNT_80_ titers are acceptably similar. The speed of the PsVNA relative to PRNT (i.e., 3 days versus up to 2 weeks for the PRNT) and the advantage of running outside of high containment make the PsVNA the logical assay choice for antibody-based product potency testing.

Polyclonal antibodies are, by definition, broadly active against multiple epitopes decreasing the chances that the infectious agent will develop drug resistance. We used pseudovirions with known MAb escape mutations to confirm that multiple epitopes spanning both the Gn and Gc proteins are targeted by the polyclonal antibody. While mapping the full repertoire of epitopes bound by the polyclonal antibodies, or the full repertoire of functional activities of the antibodies, is beyond the scope of the current study, it is clear that the purified SAB-159 and SAB-159P antibodies exhibit extremely high titer neutralizing antibody levels and levels of neutralizing antibody in serum can predict protection against infection with HTNV and PUUV. Batch to batch variability for polyclonal antibody products exist; however, the potency of the product, as measured by a functional assay such as the PsVNA, can be used to set acceptance criteria for the candidate product. Currently, MAb development involves identification of human survivors or vaccinees followed by screening for candidate molecules and the cloning of those molecules into appropriate expression systems. Using only the virus target sequence (e.g., hantavirus M gene sequence) it is possible to produce a DNA vaccine and vaccinate TcB to produce fully human anti-hantavirus antibodies. There is no need to isolate a target virus for vaccine generation and there is no need for patient involvement at any stage. The speed of vaccination, plasma collection, and antibody purification (3–5 months) and the relatively low costs (compared to monoclonal antibody development) make TcB-derived polyclonal antibodies a promising choice for emerging or rare infectious diseases.

When a monoclonal antibody is used to counter an infectious agent, such as a virus, a drawback can be the unintentional selection for mutants capable of escaping the antibody. For example, palivizumab is a monoclonal antibody used in children less than 2 years old to prevent lower respiratory tract infections caused by respiratory syncytial virus (RSV). Palivizumab escape mutants have been isolated from approximately 5% of the patients where breakthrough disease occurred after monoclonal antibody treatment (Zhu et al., 2018). In this example, there is no evidence that monoclonal antibody escape in patients resulted in worsened disease or shedding of resistant virus; however, these possibilities cannot be ruled out. To counter this, monoclonal antibody drugs are often in the form of combinations of multiple monoclonal antibodies. Two out of the three monoclonal antibody treatments for Ebola in the PALM clinical trial in the Democratic Republic of Congo are combinations of three anti-EBOV monoclonal antibodies (Sivapalasingam et al., 2018; Mulangu et al., 2019). Polyclonal antibodies such as the TcB-derived anti-HTNV and anti-PUUV described in this report, are capable of binding multiple targets and theoretically would therefore be resistant to escape mutation. Using historically identified monoclonal antibody escape mutants in HTNV G_n_ and G_c_, we demonstrated that SAB-159 was, in fact, capable of neutralizing pseudovirions engineered to escape multiple neutralizing monoclonal antibodies where “escape” was defined as a 10-fold reduction in IC_50_. If an anti-hantavirus monoclonal antibody-based product were to be developed it would likely need to either consist of a cocktail of multiple protective monoclonal antibodies binding different target sites or a single monoclonal antibody binding an epitope that cannot change. To date, an anti-hantavirus protective monoclonal antibody un-mutable epitope has not been identified.

Plasma infusion of confirmed HPS cases in Chile demonstrated a decrease in case-fatality rate with borderline statistical significance (Vial et al., 2015). That plasma trial demonstrated the promise of anti-hantavirus antibodies as treatment of hantavirus disease, but also highlighted some of the drawbacks of using human convalescent plasma or serum. For any human-derived product there is the chance of infectious agent transmission and of non-infectious risks. Routine screening for blood borne pathogens and the infectious agent of interest mitigates but does not completely abolish these risks. Non-infectious risks, such as transfusion-associated acute lung injury or allergy, must also be considered with plasma therapy (Mora-Rillo et al., 2015). Similarly, ABO blood typing is required to prevent hemolytic transfusion reactions. The limited availability of convalescent serum or plasma has an impact on dose and repeated treatments. High volumes of a standardized anti-hantavirus IgG product, such as a TcB-derived product, would eliminate the need to identify volunteers, match blood type, maintain a scalable source, and eliminate the inherent safety concerns associated with any human tissue-based product.

As no disease models for HTNV and PUUV infections exist, infection models have been characterized and are used for testing vaccines and neutralizing antibodies, with sterile immunity used as a measure of protection from infection (Hooper et al., 1999, 2001a; Brocato et al., 2013; Witkowski et al., 2017; Perley et al., 2019). Administration of low levels of SAB-159 and SAB-159P are sufficient to protect from infection, with serum neutralizing antibody titers at >100 PsVNA_50_ required to protect >90% of hamsters from infection against low-dose HTNV or PUUV challenges. Similarly, marmosets administered SAB-159 had ∼10-fold reduced levels of anti-HTNV antibody after HTNV challenge. Future studies will further refine the marmoset model to maximize its use as a primate infection model for HTNV. Passive transfer of negative control IgG from unvaccinated TcB in both the hamster and the marmoset models did not have any effect on infection demonstrating the protective efficacy of the injected human IgG is not a non-specific innate immune response to the heterologous species immunoglobulin.

The data presented here from the SAB-159 characterization experiments have implications for the use of TcB-derived IgG products in humans. Calculated from the bioavailability experiment (Figure 3A), SAB-159 has a half-life of approximately 12 days in Syrian hamsters (Figure 3B). The same concentration of SAB-159 administered 35 days prior to HTNV challenge was protective in hamsters (Figure 3C). The half-life of IgG in humans is approximately 28 days. As the TcB-derived anti- MERS CoV human IgG SAB-301 has the similar half-life as endogenous human IgG (Beigel et al., 2018). While the precise half-life of SAB-159 will need to be calculated in humans, these data suggest that SAB-159 administered at a dosage of 10 mg/kg could be bioavailable and protective in humans for > 35 days.

Until pragmatic animal models of HFRS disease are developed, it will be difficult to assess whether anti-HTNV and/or anti-PUUV can act as true therapeutics to cure disease. It is possible that, even if a disease model exists, antibody-based products alone will be insufficient to reverse disease after onset. For example, we have found that anti-ANDV antibodies produced in rabbits, non-human primates, or using TcB, can protect hamsters pre- and post-exposure to ANDV, but cannot protect after the animal has become viremic (Custer et al., 2003; Haese et al., 2015). It is possible that, for human cases, treatment with potent anti-hantavirus neutralizing antibodies combined with supportive care, and possibly in combination with broad spectrum antiviral drugs (e.g., ribavirin), will result in clinical benefit when treatment starts after disease onset. As neutralizing antibody titers correlate with disease prognosis in humans (MacNeil et al., 2010), there is a distinct advantage to early hantavirus disease treatment.

We have demonstrated previously in the ANDV/hamster disease model that neutralizing antibodies administered prior to the onset of viremia protect hamsters from lethal HPS (Hooper et al., 2008, 2014a; Brocato et al., 2012; Haese et al., 2015). The HTNV/ and PUUV/hamster infection models can be considered more rigorous models as the antiviral must prevent infection, as these viruses do not cause disease in hamsters. While viremia is not detected until Day 10 in the 10 PFU HTNV/hamster model and is undetectable in the 1,000 PFU PUUV/hamster model (Perley et al., 2019), treatment with SAB-159 or SAB-159P within 3 days is necessary to prevent infection as measured by seroconversion (Figures 5B,C). This is similar to a previous study showing the MAb HCO2 is protective and can eliminate viremia in an HFRS model when administered 4 days post infection (Liang et al., 1996). Limiting viremia by antibody passive transfer has the potential to reduce disease severity, providing further justification for anti-HFRS antibody development. It also highlights the need for an early biomarker that can be used as a trigger-to-treat. Thus far, this remains elusive.

This is the first report to show that human IgG products can be successfully generated, purified, and efficacious at preventing infection by viruses that cause HFRS in multiple animal models. The potency of these products compared to previous anti-HPS human IgG products highlights the utility of using LNP formulation for increasing vaccine immunogenicity. The proof-of-concept research described in this report lays the groundwork for future IND-enabling studies aimed at advancing anti-hantavirus human IgG products toward a Phase I clinical trial.

## Data Availability Statement

The datasets generated for this study are available on request to the corresponding author.

## Ethics Statement

The animal study was reviewed and approved by the USAMRIID Institutional Animal Care and Use Committee (IACUC).

## Author Contributions

CP, RB, HW, PC, ES, and JH conceptualized and designed the study. CP, RB, HW, CB, PK, JV, MC, BS, SK, LP, JS, and JH executed the study. HW, CB, PK, and JV provided the novel reagents and/or analytical tools. CP, RB, HW, CB, PK, JV, SK, and JH analyzed the data. CP, RB, SK, and JH wrote the manuscript. All authors contributed to manuscript revision, read, and approved the submitted version.

## Conflict of Interest

PC, ES, and JH are inventors on patents, or patents pending, related to technology used to develop proof-of-concept antibody-based products described in manuscript (i.e., lipid nanoparticles, Tc bovine, and DNA vaccines). HW, CB, and ES were employed by SAB Biotherapeutics Inc. PK, JV and PC were employed by Arcturus Therapeutics Inc. The remaining authors declare that the research was conducted in the absence of any commercial or financial relationships that could be construed as a potential conflict of interest.
