# Supplementary material for: Anti-HFRS Human IgG Produced in Transchromosomic Bovines Has Potent Hantavirus Neutralizing Activity and Is Protective in Animal Models
Source: Front Microbiol. 2020 May 7;11:832. doi: 10.3389/fmicb.2020.00832 (PMC7252588; doi:10.3389/fmicb.2020.00832)
Supplement: Supplementary file 1 [file Data_Sheet_1.docx]

**Suppl Table 1. Statistical Analyses**

| **Fig. 3C, Ordinary one-way ANOVA with Multiple Comparisons to Negative Control Group** | | | | |
| --- | --- | --- | --- | --- |
| **Group (NAU/kg)** | **n** | **Mean N-ELISA Titer (log_10_) ±SD** | **Protected/Total** | **P-value (compared to Normal TcB hu IgG group)** |
| 71,871 | 8 | 1.000 ± 0.00 | 8/8 | <0.0001 |
| 35,965 | 8 | 1.000 ± 0.00 | 8/8 | <0.0001 |
| 17,982 | 16 | 1.063 ± 0.25 | 15/16 | <0.0001 |
| 8,991 | 8 | 1.250 ± 0.46 | 6/8 | <0.0001 |
| 4,525 | 8 | 2.250 ± 0.71 | 1/8 | 0.3034 |
| 2,292 | 8 | 2.625 ± 0.52 | 0/8 | 0.9997 |
| Normal TcB hu IgG (Neg Control) | 15 | 2.667 ± 0.82 | 2/15 | ----- |
| **Fig. 3D, Ordinary one-way ANOVA with Multiple Comparisons to Negative Control Group** | | | | |
| **Day of SAB-159 Treatment** | **n** | **Mean N-ELISA Titer (log_10_) ±SD** | **Protected/Total** | **P-value (compared to Normal TcB hu IgG group)** |
| -1 | 8 | 1.000 ± 0.00 | 8/8 | <0.0001 |
| -7 | 8 | 1.000 ± 0.00 | 8/8 | <0.0001 |
| -14 | 8 | 1.250 ± 0.71 | 7/8 | <0.0001 |
| -21 | 8 | 1.000 ± 0.00 | 8/8 | <0.0001 |
| -28 | 8 | 1.000 ± 0.00 | 8/8 | <0.0001 |
| -35 | 8 | 1.500 ± 0.93 | 6/8 | 0.0007 |
| -42 | 8 | 2.125 ± 0.99 | 3/8 | 0.1878 |
| Normal TcB hu IgG (Neg Control) | 8 | 2.750 ± 0.71 | 1/8 | ----- |
| **Fig. 3E, Unpaired t-test, two-tailed, Each SAB-159 Treatment Group Compared to Same ID50** | | | | |
| **Group** | **n** | **Mean N-ELISA Titer (log_10_) ±SD** | **Protected/Total** | **P-value (compared to Same ID_50_ group)** |
| SAB-159, 5ID_50_ | 8 | 1.250 ± 0.71 | 7/8 | <0.0001 |
| Normal TcB hu IgG, 5ID_50_ (Neg Control) | 8 | 3.750 ± 0.46 | 1/8 | ----- |
| SAB-159, 25ID_50_ | 8 | 1.500 ± 0.76 | 5/8 | <0.0001 |
| Normal TcB hu IgG, 25ID_50_ (Neg Control) | 8 | 3.500 ± 0.53 | 0/8 | ----- |
| SAB-159, 50ID_50_ | 8 | 1.250 ± 0.46 | 6/8 | <0.0001 |
| Normal TcB hu IgG, 50ID_50_ (Neg Control) | 8 | 3.500 ±0.53 | 0/8 | ----- |
| **Fig. 4B, Unpaired t-test, two-tailed, SAB-159 Treated-Marmosets Compared to Negative Control Group** | | | | |
| **Group** | **n** | **Mean N-ELISA Specific OD Sum ±SD** | **Protected/Total** | **P-value (compared to Normal TcB hu IgG group)** |
| SAB-159 | 3 | 0.1337 ± 0.02 | 0/3 | 0.0078 |
| Normal TcB hu IgG (Neg Control) | 3 | 1.449 ± 0.46 | 0/3 | ----- |
| **Fig. 5A, Ordinary one-way ANOVA with Multiple Comparisons to Negative Control Group** | | | | |
| **Group (NAU/kg)** | **n** | **Mean N-ELISA Titer (log_10_) ±SD** | **Protected/Total** | **P-value (compared to Normal TcB hu IgG group)** |
| 39,676 | 8 | 1.000 ± 0.00 | 8/8 | <0.0001 |
| 19,838 | 8 | 1.000 ± 0.00 | 8/8 | <0.0001 |
| 9,919 | 8 | 1.125 ± 0.35 | 7/8 | 0.0002 |
| 4,950 | 8 | 1.500 ± 0.76 | 5/8 | 0.0378 |
| Normal TcB hu IgG (Neg Control) | 8 | 2.000 ± 0.00 | 0/8 | ----- |
| **Fig. 5B, Ordinary one-way ANOVA with Multiple Comparisons to Negative Control Group** | | | | |
| **Day of SAB-159 Treatment** | **n** | **Mean N-ELISA Titer (log_10_) ±SD** | **Protected/Total** | **P-value (compared to PBS group)** |
| D-1 | 8 | 1.000 ± 0.00 | 8/8 | 0.0009 |
| D+3 | 8 | 1.125 ± 0.35 | 7/8 | 0.0022 |
| D+5 | 8 | 1.750 ± 0.71 | 3/8 | 0.1085 |
| D+8 | 8 | 2.375 ± 0.92 | 1/8 | 0.9223 |
| PBS (Neg Control) | 8 | 2.625 ± 1.30 | 2/8 | ----- |
| **Fig. 5C, Ordinary one-way ANOVA with Multiple Comparisons to Negative Control Group** | | | | |
| **Day of SAB-159 Treatment** | **n** | **Mean N-ELISA Titer (log_10_) ±SD** | **Protected/Total** | **P-value (compared to PBS group)** |
| D-1 | 8 | 1.250 ± 0.46 | 6/8 | <0.0001 |
| D+3 | 8 | 1.750 ± 0.89 | 4/8 | 0.0014 |
| D+5 | 8 | 2.500 ± 1.07 | 2/8 | 0.1891 |
| D+8 | 8 | 3.125 ± 0.64 | 0/8 | 0.9970 |
| PBS (Neg Control) | 9 | 3.222 ± 0.67 | 0/9 | ----- |

NAU= Neutralizing antibody units

Neg= negative

ID_50_= Dose required to infect 50% of exposed animals

Hu= human

SD= standard deviation

D= Day

PRNT= plaque reduction neutralization assay

PsVNA= pseudovirion neutralization assay

**A.**

**B.**

**Suppl. Fig. 1. Bioavailability of SAB-159 in Marmosets.** A. Three marmosets were injected with a relatively low dose (2 mg/kg) of SAB-159 by the intraperitoneal route (i.p.). This is equivalent to 6,500 neutralizing antibody units (NAU)/kg. Blood was collected at the indicated timepoints and serum analyzed by HTNV PsVNA for neutralizing antibody titer. B. The data from A) was used to calculate the half-life of 7 days based on PsVNA_50_ data.
